# Effects of a 12-week gait retraining program combined with foot core exercise on morphology, muscle strength, and kinematics of the arch: A randomized controlled trial

**DOI:** 10.3389/fbioe.2022.1022910

**Published:** 2022-10-05

**Authors:** Bin Shen, Shen Zhang, Kedong Cui, Xini Zhang, Weijie Fu

**Affiliations:** ^1^ School of Exercise and Health, Shanghai University of Sport, Shanghai, China; ^2^ School of Athletic Performance, Shanghai University of Sport, Shanghai, China; ^3^ Key Laboratory of Exercise and Health Sciences of Ministry of Education, Shanghai University of Sport, Shanghai, China; ^4^ Shanghai Frontiers Science Research Base of Exercise and Metabolic Health, Shanghai University of Sport, Shanghai, China

**Keywords:** gait retraining, foot core exercises, arch morphology, arch muscles strength, arch kinematics

## Abstract

**Objective:** This study aims to explore the effects of a 12-week gait retraining program combined with foot core exercise on arch morphology, arch muscles strength, and arch kinematics.

**Methods:** A total of 26 male recreational runners with normal arch structure who used rear-foot running strike (RFS) were divided into the intervention group (INT group) and control group (CON group) (*n* = 13 in each group). The INT group performed a 12-week forefoot strike (FFS) training combined with foot core exercises. The CON group did not change the original exercise habit. Before and after the intervention, the arch morphology, as well as the strength of hallux flexion, lesser toe flexion, and the metatarsophalangeal joint (MPJ) flexors were measured in a static position, and changes in the arch kinematics during RFS and FFS running were explored.

**Results:** After a 12-week intervention, 1) the normalized navicular height increased significantly in the INT group by 5.1% (*p* = 0.027, Cohen’s *d* = 0.55); 2) the hallux absolute flexion and relative flexion of the INT group increased significantly by 20.5% and 21.7%, respectively (*p* = 0.001, Cohen’s *d* = 0.59; *p* = 0.001, Cohen’s *d* = 0.73), the absolute and relative strength of the MPJ flexors of the INT group were significantly improved by 30.7% and 32.5%, respectively (*p* = 0.006, Cohen’s *d* = 0.94; *p* = 0.006, Cohen’s *d* = 0.96); 3) and during RFS, the maximum arch angle of the INT group declined significantly by 5.1% (*p* < 0.001, Cohen’s *d* = 1.49), the arch height at touchdown increased significantly in the INT group by 32.1% (*p* < 0.001, Cohen’s *d* = 1.98).

**Conclusion:** The 12-week gait retraining program combined with foot core exercise improved the arch in both static and dynamic positions with a moderate to large effect size, demonstrating the superiority of this combined intervention over the standalone interventions. Thus, runners with weak arch muscles are encouraged to use this combined intervention as an approach to enhance the arch.

## 1 Introduction

Running is one of the most popular fitness activities today as it offers easy accessibility and obvious health gains (De Wit et al., 2000). Many researchers have investigated extrinsic and intrinsic risk factors to reduce the risk of running-related injuries (Hespanhol Junior et al., 2016; Kelly et al., 2016; Krabak et al., 2017; Yang et al., 2020). However, in the past 50 years, the rate of various lower limb injuries caused by running has remained at a relatively high level (Kakouris et al., 2021), of which the incidence of foot injuries, such as plantar fascia injury, accounted for about 20% (Taunton et al., 2002). The fascia and ligaments of the plantar are important structures that maintain the dome structure of the foot. However, maintaining the stability of the medial longitudinal arch (MLA) during gait and controlling the movement of the foot rely on the intrinsic and extrinsic foot muscles (McKeon et al., 2015). Weak foot muscles cannot provide sufficient support to MLA during dynamic movement, resulting in repeated strains of the plantar fascia, which in turn, increase the risk of plantar injuries (Cheung et al., 2016).

The anatomical structure of the longitudinal arch gives it spring-like compression and rebound characteristics, which are affected by different running postures and modern running shoes (Lieberman, 2012; Perl et al., 2012). For rearfoot strike (RFS) running, MLA rarely compresses from the foot touch down to mid-stance. In addition, impact transients associated with RFS running are sudden forces with high rates and magnitudes of loading that travel rapidly up to the musculoskeletal system, thus resulting in a high incidence of running-related injuries, especially tibial stress fractures and plantar fasciitis (Kakouris et al., 2021; Milner et al., 2006; Lieberman et al., 2010). As a result, RFS runners prefer to wear cushioned running shoes with an elastic material in the heel to reduce the transient impact forces and disperse them over time (Lieberman et al., 2010; Zhang et al., 2019). Although modern cushioned running shoes have certain shock absorption advantages, some scholars believe that the thick cushioning medium between the foot and the ground surface may damage the feedback effect of plantar mechanoreceptors (Lieberman, 2012), thus limiting arch compression and rebound, resulting in the loss of elastic work and increased metabolic energy costs (Perl et al., 2012; Stearne et al., 2016). In contrast, the arch undergoes a three-point bending during the touchdown phase of the forefoot strike (FFS) running (Perl et al., 2012). To better control arch deformation, the foot muscles will have adaptive muscle strength enhancement (Jenkins and Cauthon, 2011; Perl et al., 2012; Kelly et al., 2018). Meanwhile, the longitudinal and transverse arches include many elastic tissues that recover an estimated 17% of the mechanical energy generated per step (Ker et al., 1987). Hence, FFS can be regarded as good training to enhance the performance of the arch (Kelly et al., 2018).

Foot core exercise includes foot doming, towel curls, toe spread, and more (Lynn et al., 2012; Goo et al., 2014; Kamonseki et al., 2016). In addition, the FFS has also been proven to effectively stimulate the foot muscles and strengthen the muscle function of the foot (Kelly et al., 2018). Numerous studies have investigated the separate effects of two interventions, namely, the effect of minimal shoes or FFS on foot muscles strength or size (Miller et al., 2014; Ridge et al., 2019), and the effects of foot core exercise on the balance and postural control (Rothermel et al., 2004; Lynn et al., 2012; Mulligan and Cook, 2013). However, foot core exercise alone may fail to meet the functional requirements of the arch under dynamic conditions (e.g., running, jumping.), while FFS training alone may increase the risk of running-related injury because the foot structure cannot support the rapid increase in load caused by the sudden change in strike pattern (Ridge et al., 2013). A more appropriate training program would be a combined intervention to enhance both the intrinsic and extrinsic foot muscles (i.e., active subsystem) of the foot core system, which can simultaneously improve foot function in static posture and dynamic activities (McKeon et al., 2015). Accordingly, previous gait retraining studies have begun to incorporate foot core exercise to reduce the risk of injury in participants when the strike pattern suddenly changes (Warne et al., 2014; Deng et al., 2020; Zhang et al., 2021). Nevertheless, few studies have investigated the effect of this combined intervention on the MLA at a static position and during running. Furthermore, as the conversion of RFS to FFS is a gradual process (McCarthy et al., 2014; Zhang et al., 2020), the effects of intervention training on arch kinematics reflected in both RFS and FFS remain unclear and require further exploration.

Therefore, this study aims to investigate the effects of a 12-week gait retraining program combined with foot core exercise on arch morphology in static postures, arch muscles strength (i.e., separated toe flexion strength and the metatarsophalangeal joints (MPJ) flexor strength), and arch kinematics changes during RFS and FFS running among RFS runners. We hypothesized that the intervention would benefit arch shape maintenance, enhance arch muscle strength, and produce favorable changes in arch kinematics during running.

## 2 Methods

### 2.1 Participants

Based on the previous data (a significant group*time interaction effect on toe-flexor strength, effect size *f* = 0.6) published by Day and Hahn (2019), *a priori* power analysis (G*Power, Version 3.1.9.6, Kiel University, Germany) was conducted for expected outcomes with a type I error probability of 0.05 and an effect size *f* of 0.6 (Cohen defines effect size *f* of 0.1, 0.25, and 0.4 as small, medium, and large effects, respectively). This analysis indicated that *n* = 26 would provide a statistical power of ∼95% (Faul et al., 2007). Considering a drop-out rate of 15–20%, 32 male recreational runners were recruited through online social media, running clubs, and flyers (Wang J et al., 2020). They were all habitual runners who run at least 20 km/week in the RFS pattern while running on the ground with cushioned shoes were recruited (Lieberman et al., 2010). The inclusion criteria included the following: 1) normal arch height index (AHI, the height of the dorsum of the foot at 50% of the foot length divided by the truncated foot length) ranging from 0.31 to 0.37 (Butler et al., 2008), 2) no musculoskeletal injuries over the past 1 year and good exercise ability, 3) running distance that did not change in 3 months, and 4) never attempted foot core exercises, such as those included in this study or FFS pattern. All the participants signed an informed consent form provided by the Human Ethics Committee of Shanghai University of Sport prior to the study (IRB no. 2017007). After the baseline measurement, the participants were divided randomly into the intervention group (INT) or control group (CON). A research assistant generated the randomization schedule using a computer program and handed the results of allocation to the participants in a sealed envelope, ensuring that the researchers involved in outcome measurement and data analysis were unaware of the allocation.

### 2.2 Intervention

The INT group performed FFS training combined with foot core exercise (Tam et al., 2015; Krabak et al., 2017) to enhance intrinsic and extrinsic foot muscle strength during static and dynamic tasks. To ensure that the INT group can master the intervention method, we gathered the INT group and our running coach explained and demonstrated the FFS and foot core exercises in the first week. For the subsequent 11 weeks, the INT group underwent centralized training on an indoor track on our campus once a week. The foot core exercise protocol was based on previous literature and previous results of our team, and it had shown to be effective in enhancing foot muscles and potentially reducing the risk of injury (Warne et al., 2014; Ridge et al., 2019; Wang B et al., 2020; Zhang et al., 2021). It included heel raises, towel curls, foot doming, toe spread, balance board, and foot relaxation exercises by stepping on a tennis ball with progressive intensity (Table 1).

**TABLE 1:** Foot core exercise schedule.

	1 ∼ 2 weeks	3 ∼ 4 weeks	5 ∼ 6 weeks	7 ∼ 8 weeks	9 ∼ 10 weeks	11 ∼ 12 weeks
Heel raises (double in a flat)	1 × 10 times	2 × 10 times	2 × 15 times	—	—	—
Heel raises (double on a step)	—	—	—	2 × 20 times	2 × 25 times	3 × 30 times
Heel raises (single on a step)	—	—	—	1 × 10 times	2 × 10 times	3 × 15 times
Towel curls	3 × 10 times	3 × 20 times	3 × 10 times (+0.25 kg)	3 × 15 times (+0.25 kg)	3 × 10 times (+0.5 kg)	3 × 15 times (+0.5 kg)
Foot doming	2 × 10 times	2 × 15 times	2 × 20 times	3 × 20 times	3 × 25 times	4 × 25 times
Toe spread	2 × 10 times	2 × 15 times	2 × 20 times	3 × 20 times	3 × 25 times	4 × 25 times
Balance board	2 × 20 s	2 × 25 s	2 × 30 s	3 × 30 s	3 × 35 s	3 × 40 s
Foot relaxes	1 × 30 s	1 × 30 s	1 × 30 s	1 × 30 s	1 × 30 s	1 × 30 s

During the FFS training, each participant was required to run in an FFS pattern and was given an appropriately fitted pair of Vibram Five Fingers shoes, which aimed to protect the foot soles from the rough ground and simulate barefoot running to give the participant a habituation process for the strike pattern transition (Paquette et al., 2013; McCarthy et al., 2014). The duration of FFS running was increased every week based on protocols from previous literature (Tam et al., 2015; Zhang et al., 2021) (Table 2). The INT group was allowed to do rear-foot strike running with cushioning running shoes when out of training to maintain a running distance of a total of at least 20 km/week.

**TABLE 2:** FFS training schedule.

Week	1	2	3	4	5	6	7	8	9	10	11	12
Duration (min)	5	10	15	20	25	30	35	40	42	44	46	48
Times per week	3	3	3	3	3	3	3	3	3	3	3	3

Participants in the CON group were required to continue their regular running routines in an RFS pattern using cushioned running shoes and running at their habitual pace in places where they were accustomed to running (e.g., parks, roads, tracks.) for 12 weeks. All participants in the INT and CON groups were required to provide us feedback after each training, including running location, duration, and mileage, as well as photos while doing foot core exercises. During the entire experiment and intervention, no participants suffered injuries.

The criteria for successful intervention were as follows: 1) completion of all the tests, 2) no more than three times of absences, and 3) completion of the last three-week training. During the intervention period, the participants were allowed to postpone or quit due to injuries or personal reasons (Zhang et al., 2020).

### 2.3 Arch morphology

**FIGURE 1:**
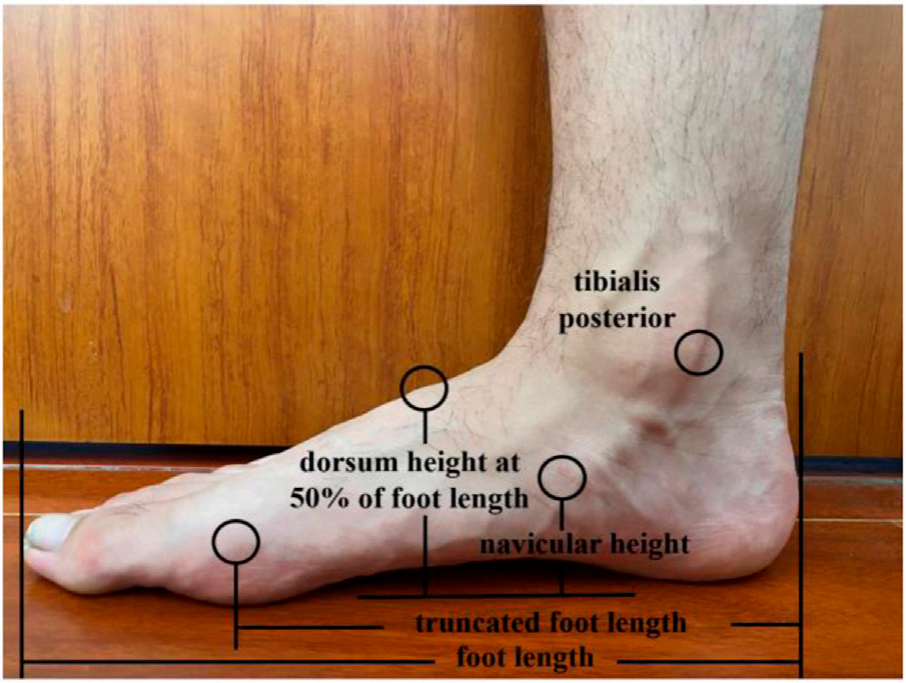
Arch morphology variables.

Initially, a participant was in a standing posture, and the most prominent aspect of the navicular tuberosity was palpated by a certified rehabilitation therapist who performed all of the palpation, manual measurements, and marker sticking throughout the whole experiment (Langley et al., 2016). If the navicular tuberosity was not sufficiently prominent, we instructed the subject to maintain the foot adducted and identified the navicular tuberosity by palpation along the clearly visible tendon of the posterior tibialis (Golano et al., 2004). The position of navicular tuberosity was marked with a washable pen for subsequent measurements. Then, a digital caliper was used to test the arch morphology in the standing and sitting positions in this study. In the standing position, participants were required to keep upright and maintain a bilateral standing posture with their bare feet pelvis-width apart as they tried to make their legs as parallel as possible to avoid foot inversion or eversion. When switching to a sitting position, the participants sat back in the seat without moving their feet and kept their hip, knee, and ankle joints in 90° flexion, as well as the hands hanging at the sides of the body. Then, both forefeet were raised and lowered on the ground with subtalar joint in a natural position. The arch morphological variables (Williams and Mcclay, 2000; Fiolkowski et al., 2003) included the following: 1) standing/sitting arch height: distance from navicular center to the ground; 2) normalized arch height: navicular height divided by foot length; and 3) navicular drop: the difference between standing arch height and sitting arch height (Figure 1).

### 2.4 Arch muscles strength

#### 2.4.1 Separated toe flexion strength

**FIGURE 2:**
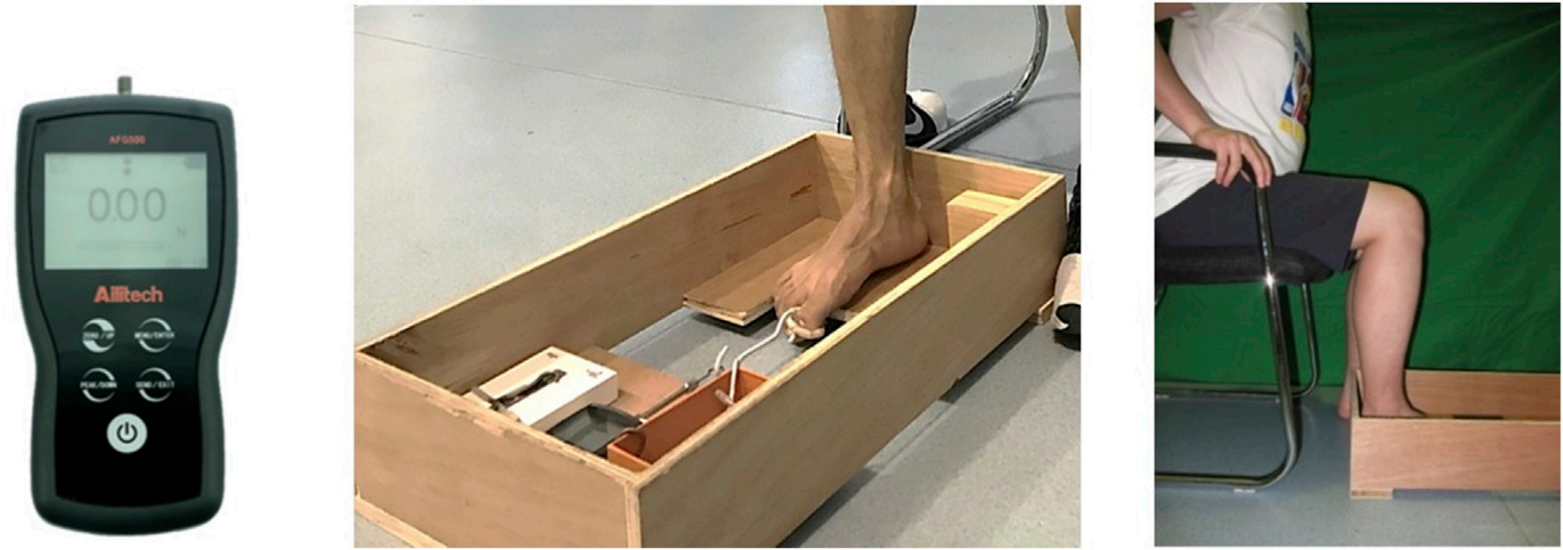
The digital calliper and separated toes flexion test (Zhang et al., 2019).

A modified dynamometer (Ailitech ADF 500, China) attached to a wooden frame (Figure 2) was used to measure the hallux flexion initially, and then simultaneously with the lesser toe flexion (from 2nd to 5th toes). The board under the foot can provide support from the heel to the head of the first metatarsal, thereby allowing toe flexion. To avoid missing the force values, metal hooks and rings were used to connect the toes and the dynamometer. During the test, participants were required to maintain a sitting position with the hip, knee, and ankle joints flexed at 90°.

To test hallux flexion, the hallux was aligned with the dynamometer and then a metal ring was set to the hallux. When the hallux relaxed, the force value was 0 N. The participants were required to flex their big toe and pull the ring back as hard as possible. The tests were repeated if the participants’ heel and/or ball of the foot left the board. The combined strength of the 2nd to 5th toes was assessed in a similar manner. This method has been associated with excellent repeatability and reliability for all tests between days, raters, and sessions (Ridge et al., 2017; Zhang et al., 2019).

#### 2.4.2 Metatarsophalangeal joint flexors strength

**FIGURE 3:**
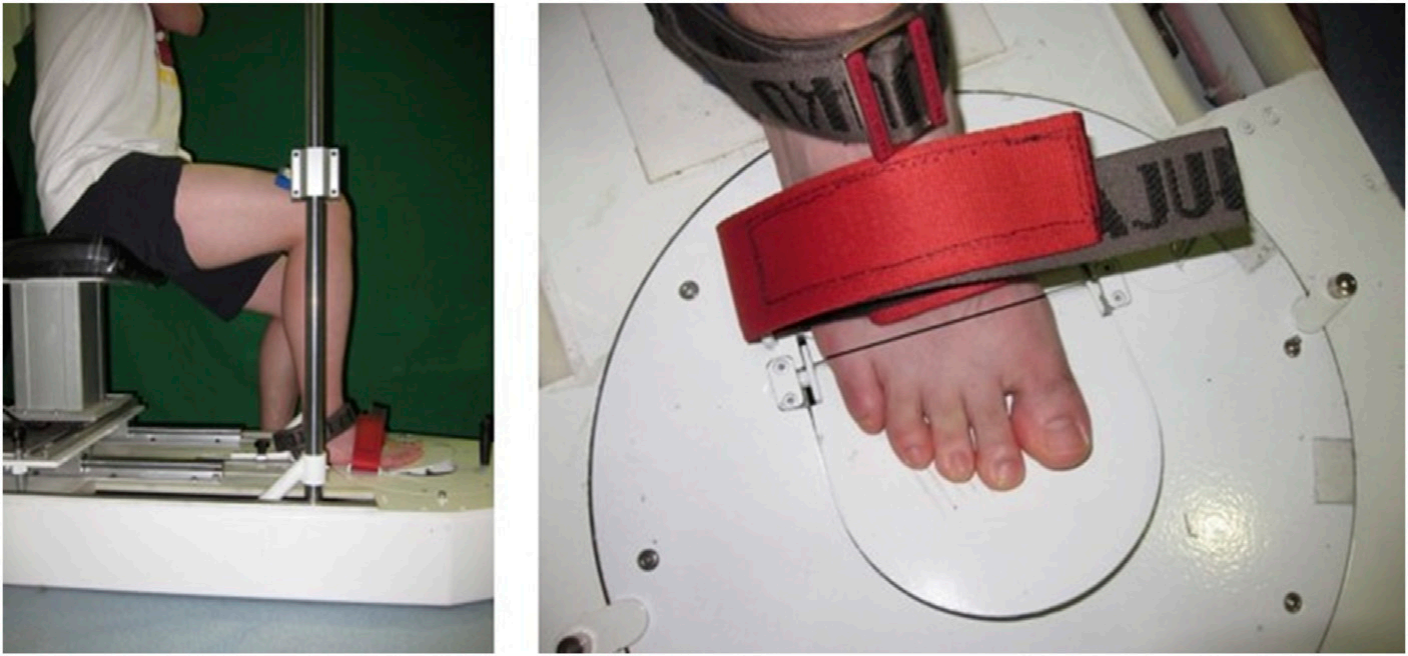
A self-developed strength tester for the metatarsophalangeal joint flexors (Zhang et al., 2019).

A customized strength tester (CN103278278B, China) was used to measure the MPJ flexors strength with the sampling rate of 120 Hz. The strength tester consisted of a chassis, a seat, force sensors, a computer, a foot platform, and a 30° raised toe platform, and was similar to the device used in the study of Man et al. (2016). The participants were required to sit on a chair with their knees and feet fixed by a stopper plate to avoid the data being interfered with by other joints. During the tests, they were asked to keep the toes as close to the surface of the toe platform as possible and then try their best to flex all their MPJs together for 10 s to press the toe platform. If there was an elevation of the interphalangeal joints, the test was considered a failure and then repeated after a short rest. The tester used the pedal movement is transmitted the metatarsophalangeal by the tension sensor (Figure 3). The MPJ flexor strength data were measured and processed using a computer. The interclass correlation coefficients (ICC = 0.874) of this measurement were calculated in SPSS to test the reliability between sessions of the same rater on the same day (Ridge et al., 2017; Zhang et al., 2019).

### 2.5 Arch kinematics and arch stiffness

**FIGURE 4:**
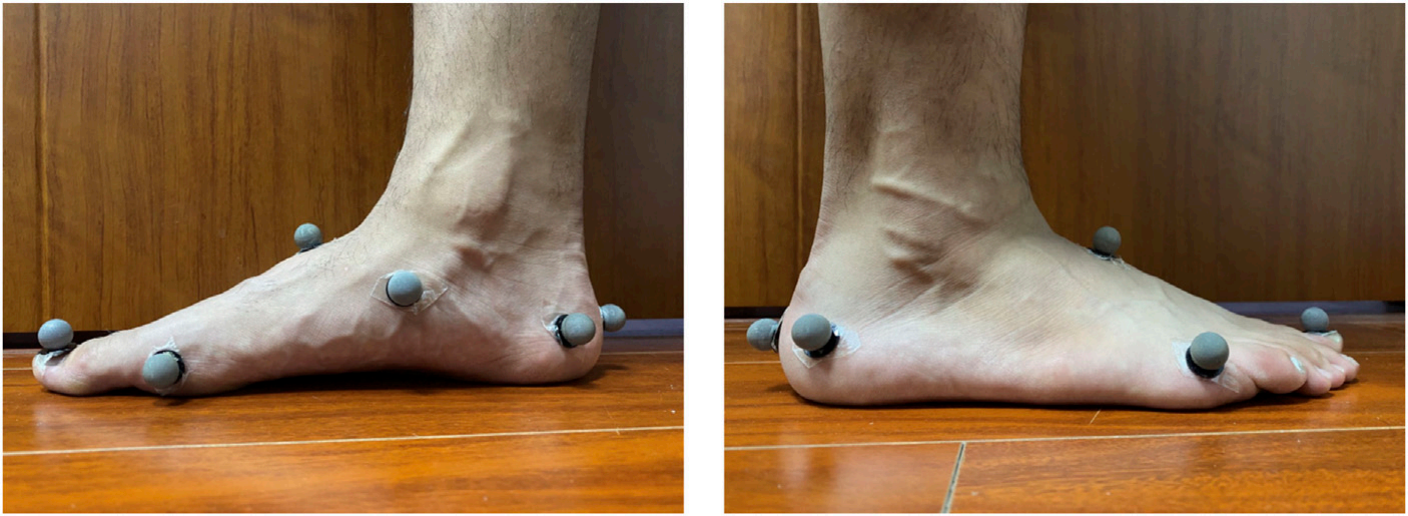
Marker position.

A 12-camera motion analysis system (100Hz, Vicon Motion Analysis Inc., Oxford, United Kingdom) was used to obtain the three-dimensional (3D) kinematic data of the foot. Given the methodological design described in the previous study, more accurate data on the arch kinematics of participants were obtained by placing infrared reflective markers onto the dominant leg at the following landmarks in the barefoot condition (Perl et al., 2012): 1) the first toe, 2) the medial side of the first metatarsal joint, 3) the lateral side of the 5th metatarsal head, 4) the navicular tuberosity, 5) the highest point on the dorsum, 6) the medial calcaneus process, 7) the lateral calcaneus process, and 8) the location of Achilles tendon insertion on the calcaneus (Figure 4). Two 90 × 60 × 10 cm force platforms (1000 Hz, 9287 B, Kistler Corporation, Winterthur, Switzerland) were used to collect ground reaction force (GRF) simultaneously with 3D kinematic data using the Vicon workstation during running.

The INT and CON groups were assessed using RFS and FFS barefoot running tests pre- and post-intervention. We introduced the RFS pattern to the participants, which was defined as initial heel contact, and the FFS pattern as initial forefoot contact much like “running on one’s toes” (Williams et al., 2012; Rice and Patel, 2017). Before the running tests, the participants performed a warm-up protocol consisting of 5 min on the treadmill at a self-selected speed running followed by a set of static stretching exercises. At the start of the recording sessions, the participants were instructed to practice running barefoot across the running path until they could touchdown naturally. Three successful trials were recorded while their right foot comes into contact with the force plate at a speed of 3.33 m/s (±5%) (McCarthy et al., 2014).

Kinematic data and GRF were analyzed *via* the Visual 3D (V5, C-Motion, Inc., Germantown, MD, United States). All marker trajectories were filtered using a fourth-order Butterworth low-pass filter with a cut-off frequency of 7 Hz. The GRF was filtered with a cut-off frequency of 50 Hz (Kelly et al., 2018). The ankle joint rotation was calculated *via* Cardan sequencing where motion about the X-axis was defined as plantarflexion/dorsiflexion (Williams et al., 2012).

**FIGURE 5:**
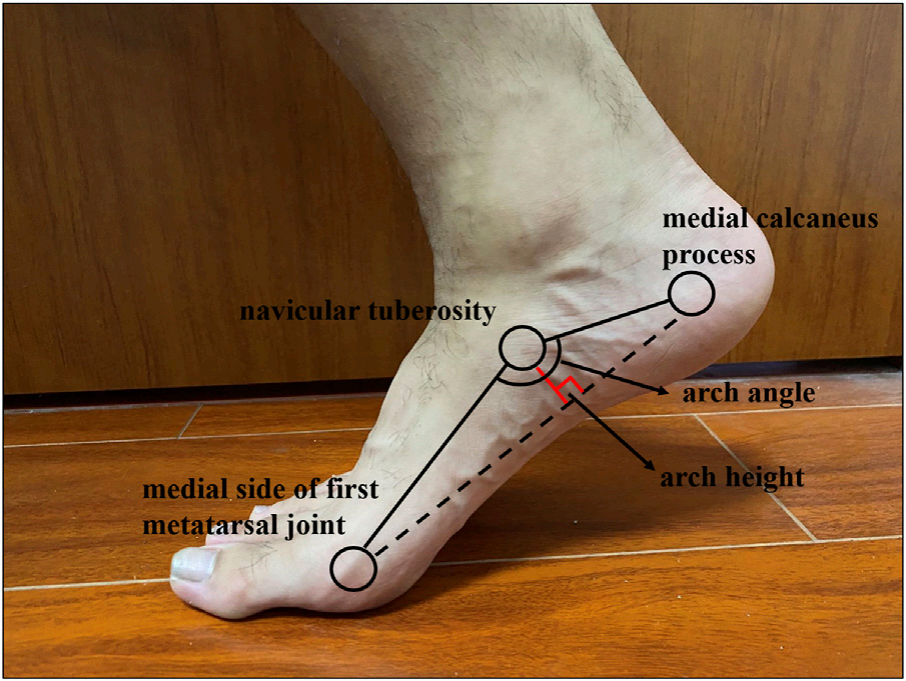
Arch kinematic variables.

Arch kinematic variables included the following (Holowka et al., 2018): 1) Δarch angle, which is the angle variation of the metatarsal bone relative to the calcaneus from touchdown to the maximum angle; 2) maximum arch angle, which is the arch angle compressed to maximum value during the stance phase; 3) arch height at touchdown, which is the perpendicular distance at touchdown between the navicular tuberosity and a line bisecting the medial side of the first metatarsal head and the medial calcaneus process; and 4) Δarch height, which is the difference between arch height at touchdown and arch height at 50% of stance phase 5) arch stiffness, the change in arch height dividing the vertical GRF at mid-stance normalized by (body mass)^0.67^ (Figure5).

### 2.6 Statistics

The results are shown with mean and standard deviation for each variable. All variables were inspected for normality and for homogeneity of variance between the two groups. Arch stiffness was log-transformed to achieve normality (Holowka et al., 2018). A 2 × 2 mixed factor ANOVA with baseline data as a covariate was used to examine the interaction effects of groups (INT vs. CON) and time (before vs. after intervention) on the arch morphology, arch muscle strength, and arch kinematics variables (Wang et al., 2021). When a significant interaction effect or main effect was detected, independent *t*-tests and paired *t*-tests were used as a post-hoc test to compare differences between-groups and within-subjects, respectively. When a significant main effect of time was detected, independent *t*-tests were used to compare the differences in percentage changes between the two groups. Effect sizes were calculated for ANOVA and *t*-test comparisons using $\eta_{p}^{2}$ and Cohen’s *d* respectively. $\eta_{p}^{2}$ of 0.01, 0.09, and 0.25 were respectively interpreted as small, medium and large effects, while Cohen’s *d* of 0.2–0.5, 0.5–0.8 and >0.8 were respectively interpreted as small, moderate and large effects (Miller et al., 2014; Wang et al., 2021). The level of significance was set at *α* = 0.05. If no significant changes were found, the Cohen’s *d* was calculated and reported. All statistical analyses were performed in SPSS (19.0, SPSS Inc., Chicago, IL, U.S.A.).

## 3 Results

### 3.1 Dropout rate

**FIGURE 6:**
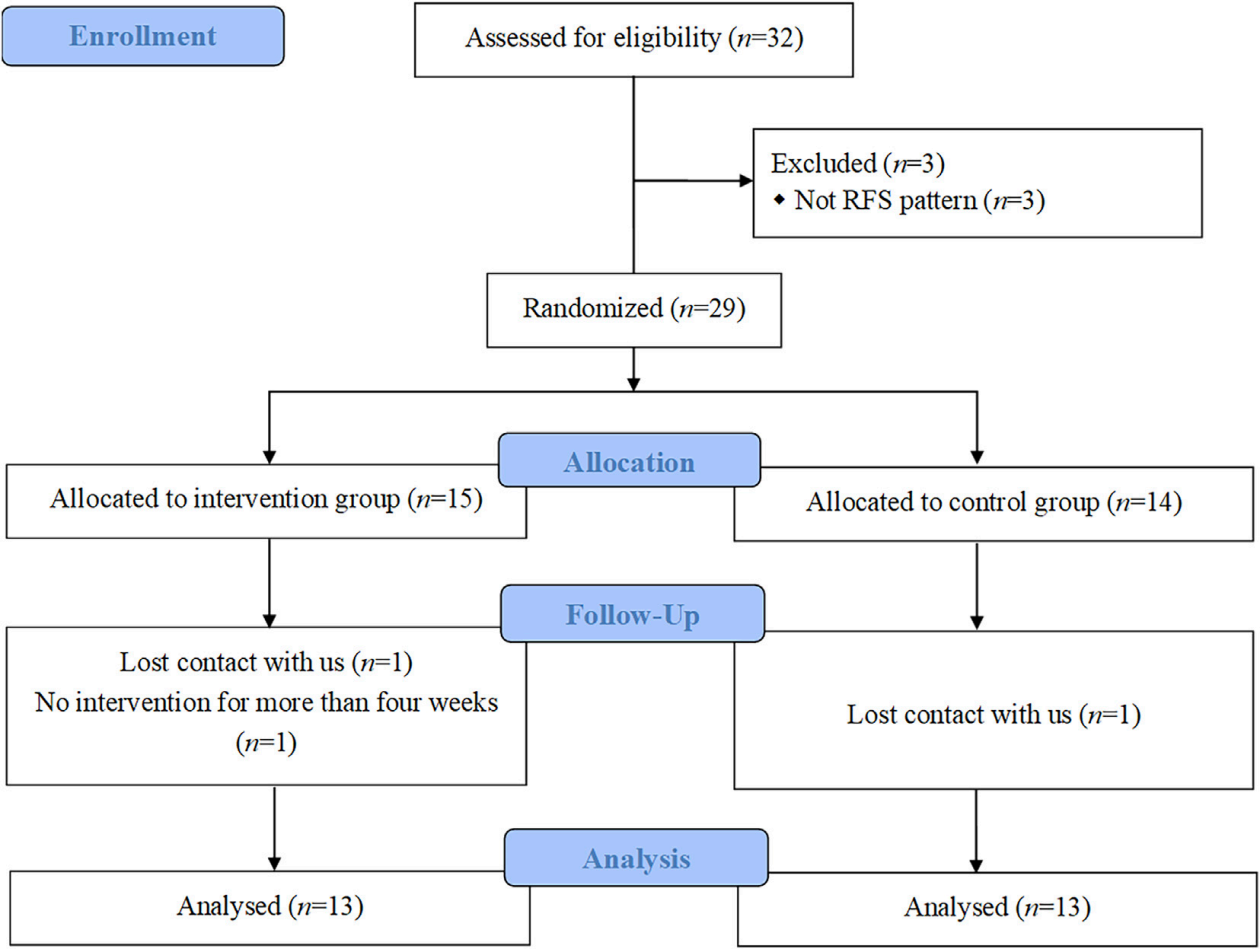
CONSORT flow chart.

Six participants dropped out. Among them, three FFS participants were excluded from the test before training due to their inability to meet the inclusion criteria, one participant did not train for more than 4 weeks due to a business trip, and two participants lost contact with us. Given that the per-protocol approach has been widely used in previous studies on gait retraining or foot muscle training (Miller et al., 2014; Ridge et al., 2019), which could be because including participants who did not complete the intervention program in the final analysis would underestimate the potential benefits of the intervention (Moher et al., 2010). Based on the above consideration, we also used a per-protocol approach to analyse all outcomes. Thus, 26 participants who completed the intervention and met the inclusion criteria (13 in the INT, 13 in the CON) were included in the final analysis and statistics (Table 3; Figure 6).

**TABLE 3:** The basic information of participants.

Group	Age (yrs)	Height (cm)	Weight (kg)	AHI	Running distance (km/w)	Training frequency (d/w)
INT (*n* = 13)	25.2 ± 4.8	175.5 ± 8.2	72.8 ± 14.9	0.34 ± 0.02	29.4 ± 4.3	2.8 ± 0.4
CON (*n* = 13)	23.8 ± 1.7	176.6 ± 4.9	72.0 ± 7.1	0.35 ± 0.01	28.8 ± 3.0	2.8 ± 0.4

### 3.2 Arch morphology

**FIGURE 7:**
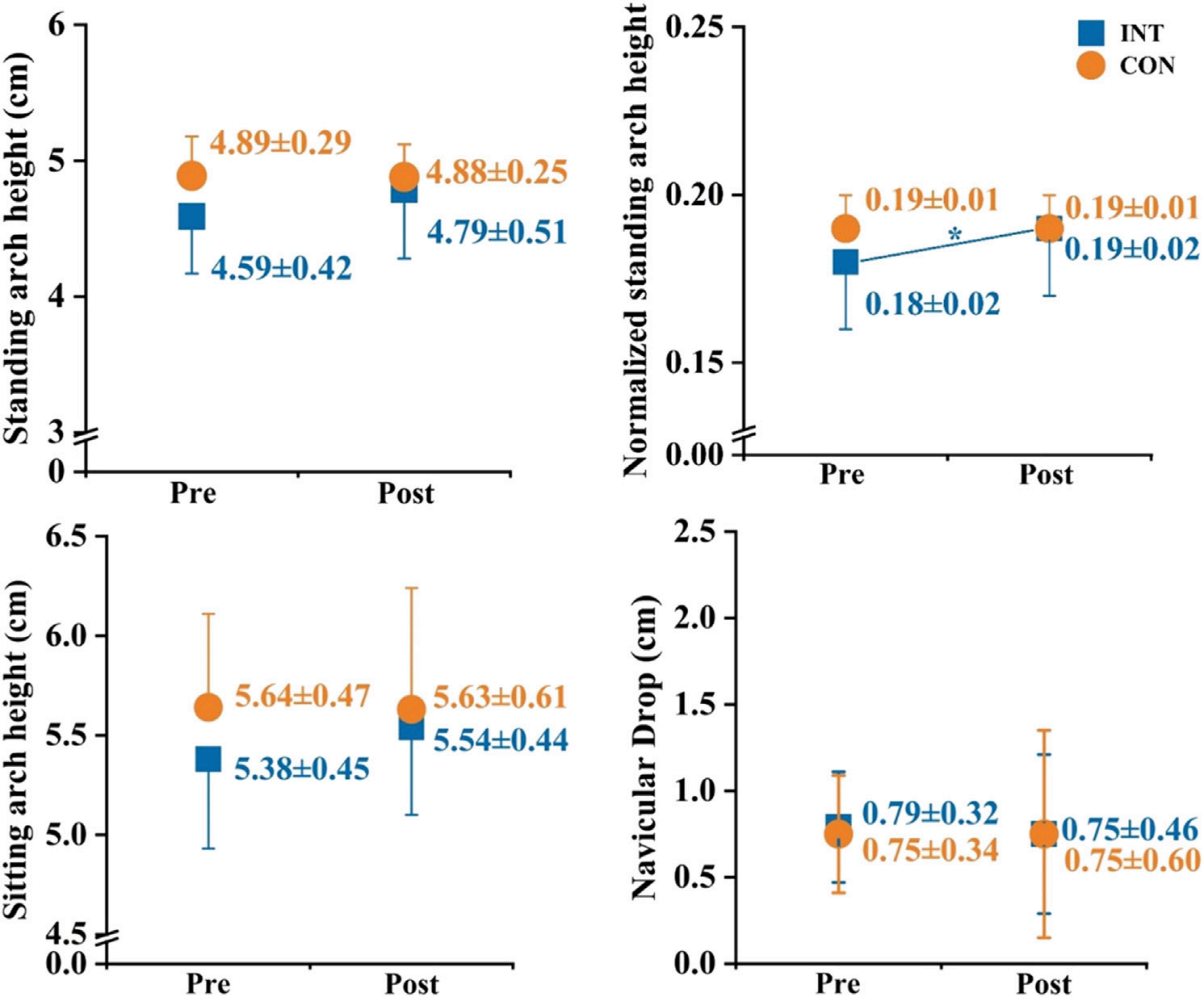
Effects of a 12-week gait retraining program combined with foot core exercise on the arch morphology. Notes: * significant difference from pre- to post-tests.

A significant main effect of time on the normalized standing arch height was observed ( $F_{1,23}$ = 6.430, *p* = 0.018, $\eta_{p}^{2}$ = 0.218). Paired *t*-tests indicated that after the intervention, the normalized standing arch height increased significantly in the INT group by 5.1% (*p* = 0.027, Cohen’s *d* = 0.55) while decreased in the CON group by 1.6% (*p* = 0.427, Cohen’s *d* = 0.18), and independent *t*-tests showed a significant difference in percentage changes between the INT group and the CON group (*p* = 0.025, Cohen’s *d* = 0.94). There was no significant interaction effect or main effect on standing/sitting arch height and navicular drop (Figure 7).

### 3.3 Arch muscles strength

#### 3.3.1 Separated toe flexion strength

Significant interaction effects between time and group on the hallux absolute flexion ( $F_{1,23}$ = 5.840, *p* = 0.024, $\eta_{p}^{2}$ = 0.202) and the hallux relative flexion ( $F_{1,23}$ = 4.974, *p* = 0.036, $\eta_{p}^{2}$ = 0.178) were observed. Paired *t*-tests revealed that after the intervention, the hallux absolute flexion and relative flexion of the INT group increased significantly by 20.5% and 21.7%, respectively (*p* = 0.001, Cohen’s *d* = 0.59; *p* = 0.001, Cohen’s *d* = 0.73), while the CON group showed no significant changes (*p* = 0.842, Cohen’s *d* = 0.04; *p* = 0.762, Cohen’s *d* = 0.06). There was no significant interaction effect or main effect on lesser toe absolute flexion and lesser toe relative flexion.

#### 3.3.2 Metatarsophalangeal joint flexors strength

**FIGURE 8:**
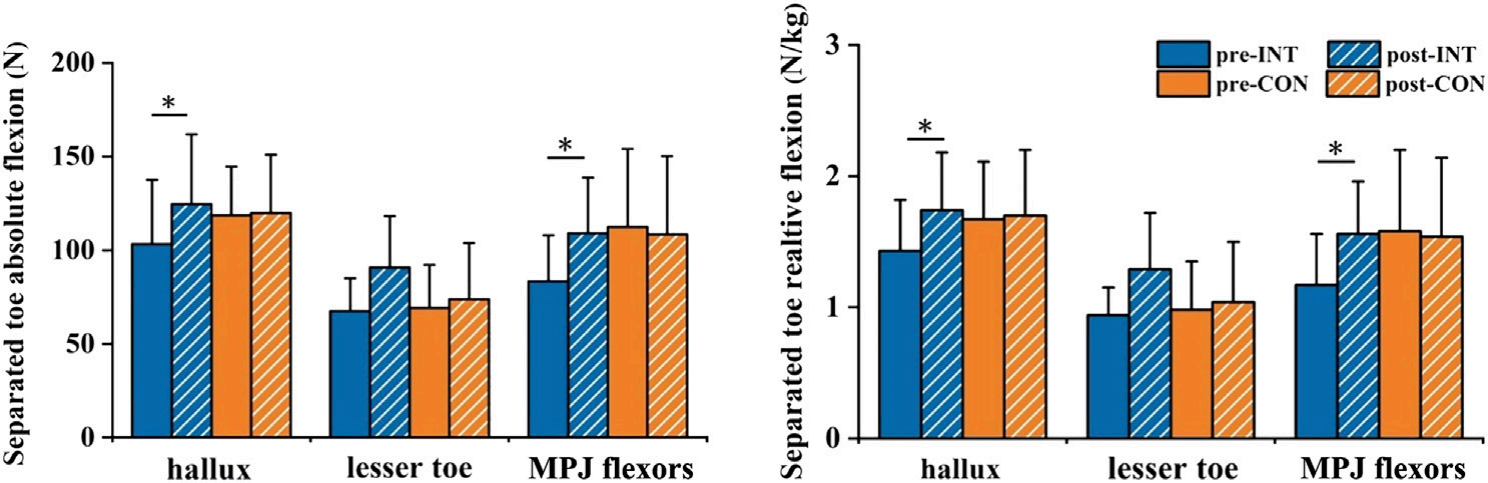
Effects of a 12-week gait retraining program combined with foot core exercise on arch muscles strength. Notes: * significant difference from pre- to post-tests.

There were significant main effects of time on the absolute ( $F_{1,23}$ = 9.399, *p* = 0.005, $\eta_{p}^{2}$ = 0.290) and relative strength ( $F_{1,23}$ = 11.785, *p* = 0.002, $\eta_{p}^{2}$ = 0.339) of the MPJ flexors. Paired *t*-tests revealed that after the intervention, the absolute and relative strength of the MPJ flexors increased significantly in the INT group by 30.7% and 32.5%, respectively (*p* = 0.006, Cohen’s *d* = 0.94; *p* = 0.006, Cohen’s *d* = 0.96), while no significant changes in the CON group (*p* = 0.749, Cohen’s *d* = 0.10; *p* = 0.813, Cohen’s *d* = 0.07). (Table 4; Figure 8).

**TABLE 4:** Effects of a 12-week gait retraining program combined with foot core exercise on the arch muscles strength.

Variables	INT		CON	
	Pre	Post	Pre	Post
Hallux absolute flexion (N)	103.31 ± 34.38	124.55 ± 37.25^*^	118.65 ± 25.97	119.86 ± 31.00
Hallux relative flexion (N/kg)	1.43 ± 0.39	1.74 ± 0.44^*^	1.67 ± 0.44	1.70 ± 0.50
Lesser toe absolute flexion (N)	67.52 ± 17.58	90.79 ± 27.39	69.24 ± 22.95	73.77 ± 30.04
Lesser toe relative flexion (N/kg)	0.94 ± 0.21	1.29 ± 0.43	0.98 ± 0.34	1.05 ± 0.46
Metatarsophalangeal joint flexors absolute strength (N)	83.47 ± 24.54	109.08 ± 29.71^*^	112.44 ± 41.57	108.36 ± 41.89
Metatarsophalangeal joint flexors relative strength (N/kg)	1.17 ± 0.39	1.55 ± 0.40^*^	1.58 ± 0.60	1.54 ± 0.60

* significant difference from pre- to post-tests.

### 3.4 Arch kinematics and arch stiffness

All participants exhibited consistent ankle dorsiflexion at touchdown in RFS (INT vs. CON in the pre-test: 6.74 ± 4.29 vs. 5.05 ± 4.38; INT vs. CON in the post-test: 7.46 ± 4.10 vs. 6.52 ± 4.59), and ankle plantarflexion at touchdown in FFS (INT vs. CON in the pre-test: –14.65 ± 5.81 vs. –15.48 ± 4.31; INT vs. CON in the post-test: –12.70 ± 6.54 vs. –14.59 ± 3.83).

During RFS, there was a significant interaction effect between time and group on the maximum arch angle ( $F_{1,23}$ = 5.296, *p* = 0.031, $\eta_{p}^{2}$ = 0.187), paired *t*-tests revealed that after the intervention, the maximum arch angle of the INT group declined significantly by 5.1% (*p* < 0.001, Cohen’s *d* = 1.49), while the CON group showed no significant change (*p* = 0.733, Cohen’s *d* = 0.08). The significant main effects of time were observed on Δarch angle ( $F_{1,23}$ = 6.885, *p* = 0.015, $\eta_{p}^{2}$ = 0.230), the arch height at touchdown ( $F_{1,23}$ = 41.658, *p* < 0.001, $\eta_{p}^{2}$ = 0.644), Δarch height ( $F_{1,23}$ = 13.949, *p* = 0.001, $\eta_{p}^{2}$ = 0.378), and arch stiffness ( $F_{1,23}$ = 31.030, *p* < 0.001, $\eta_{p}^{2}$ = 0.574). Paired *t*-tests indicated that after the intervention, the arch height at touchdown and the Δarch height increased significantly in the INT group by 32.1% and 29.5%, respectively (*p* < 0.001, Cohen’s *d* = 1.98; *p* = 0.023, Cohen’s *d* = 0.71), and the arch stiffness decreased significantly in the INT group by 33.3% (*p* = 0.021, Cohen’s *d* = 0.80), while no significant changes in the CON group (*p* = 0.811, Cohen’s *d* = 0.08; *p* = 0.620, Cohen’s *d* = 0.09; *p* = 0.210, Cohen’s *d* = 0.42). Independent *t*-tests showed a significant difference in percentage changes between the INT group and the CON group on the arch height at touchdown (*p* < 0.001, Cohen’s *d* = 1.80).

**FIGURE 9:**
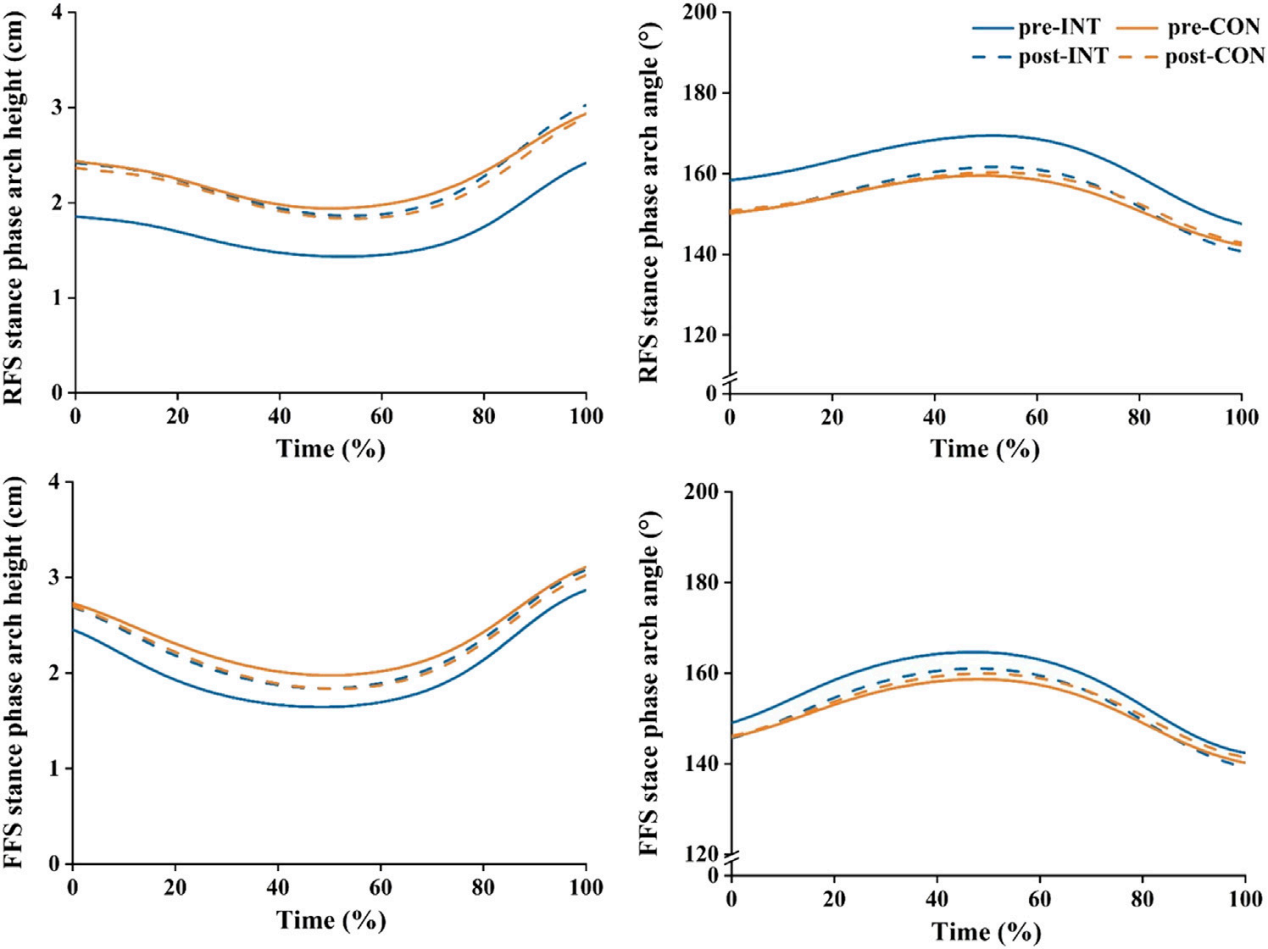
Effects of a 12-week gait retraining program combined with foot core exercise on the arch height and angle.

During FFS, the significant main effects of time were observed on Δarch angle ( $F_{1,23}$ = 15.411, *p* = 0.001, $\eta_{p}^{2}$ = 0.401), the maximum arch angle ( $F_{1,23}$ = 221.248, *p* < 0.001, $\eta_{p}^{2}$ = 0.906), the arch height at touchdown ( $F_{1,23}$ = 77.381, *p* < 0.001, $\eta_{p}^{2}$ = 0.771), Δarch height ( $F_{1,23}$ = 13.565, *p* = 0.001, $\eta_{p}^{2}$ = 0.371), and arch stiffness ( $F_{1,23}$ = 20.507, *p* < 0.001, $\eta_{p}^{2}$ = 0.471). Paired *t*-tests indicated that after the intervention, the arch height at touchdown increased significantly in the INT group (*p* = 0.040, Cohen’s *d* = 0.81), while no significant changes in the CON group (*p* = 0.845, Cohen’s *d* = 0.08) (Table 5; Figure 9).

**TABLE 5:** Effects of a 12-week gait retraining program combined with foot core exercise on the arch kinematics.

Variables	INT		CON	
	Pre	Post	Pre	Post
RFS				
ΔArch angle (°)	11.29 ± 1.47	11.22 ± 2.26	9.69 ± 3.74	9.60 ± 2.79
Maximum arch angle (°)	170.07 ± 6.51	161.45 ± 5.00*	160.58 ± 5.47	160.20 ± 4.63
Arch height at touchdown (cm)(cm)	1.84 ± 0.30*#	2.43 ± 0.30*	2.44 ± 0.44	2.41 ± 0.24
ΔArch height (cm)	0.44 ± 0.19	0.57 ± 0.17*	0.52 ± 0.21	0.54 ± 0.18
Arch stiffness (N/cm·kg^2/3^)	266.47 ± 142.63	177.68 ± 64.47*	269.20 ± 210.21	203.77 ± 71.77
FFS				
Δ Arch angle (°)	15.47 ± 2.22	15.59 ± 2.05	12.14 ± 3.04	13.59 ± 2.73
Maximum arch angle (°)	169.48 ± 18.27	161.02 ± 4.72	158.40 ± 7.41	159.92 ± 4.00
Arch height at touchdown (cm)(cm)	2.47 ± 0.33	2.72 ± 0.30*	2.71 ± 0.56	2.68 ± 0.20
Δ Arch height (cm)	0.81 ± 0.19	0.87 ± 0.19	0.76 ± 0.21	0.84 ± 0.19
Arch stiffness (N/cm·kg^2/3^)	127.51 ± 30.25	119.15 ± 30.10	161.74 ± 76.30	132.75 ± 30.47

* significant difference from pre- to post-tests.

## 4 Discussion

The purpose of this study was to investigate the effect of a 12-week gait retraining program combined with foot core exercise on arch morphology and arch muscle strength, and to further explore changes in arch kinematics in the RFS and FFS patterns. Overall, 12-week gait retraining combined with foot core exercise improved the arch height when standing, strengthen the toe flexors, decrease the maximum arch angle, and increase the arch height at touchdown during RFS running. Furthermore, the proposed intervention caused adaptive changes in the arch and surrounding muscles, which were also reflected in the arch kinematic of the running gait.

### 4.1 Arch morphology

The 12-week gait retraining combined with foot core exercise significantly increased the normalized standing arch height in the INT group, thus corroborating the finding of McKeon et al. (McKeon et al., 2015). Moreover, the improvement reached a moderate effect size (Cohen’s *d* = 0.55), which was superior to previous studies with no significant changes in arch height after standalone interventions (Lynn et al., 2012; Miller et al., 2014). Some researchers pointed out that the function of the foot intrinsic muscles is directly related to the structure of the longitudinal arch (Fiolkowski et al., 2003; Soysa et al., 2012; Kelly et al., 2014; Xiao et al., 2020). For example, to prevent the occurrence of excessive arch deformation, the recruitment of foot muscles increases with external increasing load when a decrease in electromyographic activity of these muscles leads to an increase in the navicular drop, thus revealing the relationship between the lower extremity loading and the foot muscles activation levels (Fiolkowski et al., 2003; Kelly et al., 2014). Accordingly, the shape of the arch could be stabilized by strengthening the foot muscles using the rowel curls, foot doming, and toe spread, which were proven to be effective interventions to strengthen the arch muscles (Jung et al., 2011; Yoo, 2014; McKeon et al., 2015). In addition, forefoot strike running with minimal shoes can stimulate the arch muscle function development in the activity due to the lack of plantar support, which will increase the work requirements of the foot and ankle muscles (Altman and Davis, 2012). For example, an intervention comprising 12-week forefoot running in minimal shoes increased the anatomical cross-sectional area and muscle volume of flexor digitorum brevis and abductor digiti minimi (Miller et al., 2014). Studies have also shown that the cross-sectional area is proportional to the maximum strength of the muscle (An et al., 1991). Given a number of studies that the foot intrinsic and extrinsic muscles are effective in maintaining the arch shape, the 12-week intervention method in this research can strengthen the function of the foot muscles and improve the arch shape under the condition of weight-bearing.

### 4.2 Arch muscles strength

After the 12-week gait retraining combined with foot core exercise, the strength of hallux flexion and the MPJ flexors improved effectively. It is worth noting that the improvements in arch muscles strength all had a moderate to large effect size, with the percentage change in relative strength of the MPJ flexors reaching 32.5%, which was higher than that achieved by Day and Hahn (2019) who used foot muscle training alone (27%). The plantar intrinsic muscles include abductor hallucis, flexor digitorum brevis and quadratus plantae as accessory toe flexors (Kelly et al., 2014), which produce force at the MPJ (Koyama et al., 2019). In the single-support phase and early stage of double support phase in the gait, the metatarsophalangeal moment plays a role in controlling the angular momentum of the whole body, with the maximum metatarsophalangeal moment being as large as one-fifth to one-third of the maximum ankle joint plantarflexion moment (Miyazaki and Yamamoto, 1993). Accordingly, the toe flexion and extension activities are particularly important in running and jumping, which need the action to push off. Therefore, many coaches of sprint and jumping events believe that increasing the output power of the MPJ can improve athletes’ sports performance (Goldmann et al., 2013).

These muscles that affect the strength of the MPJ are also necessary structures to support the longitudinal arch. A previous study has provided evidence that strengthening these muscles can attenuate the mechanical load directly related to running-related injuries, with the result that participants in the foot core training group had a 2.42-fold lower rate of running-related injuries compared with the control group (Taddei et al., 2020). In our research, an intervention method combining FFS training and foot core exercise was used to try to strengthen the arch muscles’ ability to exert force in a relatively static and running gait. Some of our findings confirm the hypothesis of this research, therefore, our methods can be referenced by athletes in running and jumping events to obtain stronger foot muscle function.

### 4.3 Arch kinematics and arch stiffness

During RFS, the 12-week gait retraining combined with foot core exercise reduced the maximal arch angle by 5.1% while increasing the arch height at touchdown by 32.1%. However, Day and Hahn (2019) showed that foot muscle training alone had no effect on the mechanics of the MPJ and ankle during running, suggesting that kinematic changes in this study may benefit from additional effects of the combined intervention. When a certain type of exercise forms a repetitive load on the body, special neuromuscular adaptability will appear, such as the sport-specific adaptability of foot muscle function and foot structure (Häkkinen and Keskinen, 1989; Koyama et al., 2019). In the late swing of FFS, the abductor hallucis activation is increased, resulting in a higher arch at touchdown to allow the midfoot to compress a larger range of motion during the stance phase without altering arch peak deformation (McDonald et al., 2016; Kelly et al., 2018). Considering the abovementioned results of arch muscle strength, the foot training methods in this research effectively enhanced the arch muscles, and the arch kinematic performance in running was improved to a certain extent. Furthermore, when considering how to increase the foot-spring function during the gait, Perl et al. (Perl et al., 2012) found that at the same running speed, the FFS pattern has higher arch compliance compared to RFS, which means a greater range of midfoot motion. As the foot intrinsic muscles and tendons have relatively long tendons, they can be stretched and shortened during the running stance phase, thus allowing the storage of energy in the tendons. The resulting higher compliance may be very conducive to elastic energy storage and release, thus reducing the elastic potential energy loss in this process (Kelly et al., 2018). This research found that after intervention training, RFS showed similarities in arch movement characteristics with the FFS, namely, the arch height increased at touchdown, and the motion of the midfoot increased. However, whether the increase in the motion of the midfoot during the RFS stance phase is conducive to the advancement of the body may require further analysis.

The arch stiffness decreased in the post condition during RFS, it may be that the participants gradually mastered a more comfortable running gait during multiple familiarization sessions prior to the test to reduce the peak impact in the RFS (Altman and Davis, 2012; Thompson et al., 2015). Furthermore, the greater decrease in stiffness in the INT group (33.3%) than in the CON group (24.3%) may benefit from the increased compliance of the arch due to the 12-week gait retraining program combined with foot core exercise (Kelly et al., 2018).

In summary, the 12-week gait retraining combined with foot core exercise in this study had certain effects on the arch morphology, arch muscle strength, and arch kinematics characteristics of RFS. As an important part of the lower limb system, whether the changes in the arch kinematics during the RFS are beneficial to the health of the foot may need further exploration.

There are several limitations to this study: 1) we did not continue to observe the adaptive changes of arch function in different running postures to judge the durability of the intervention; 2) arch dynamics were not analyzed in this study, which may further explore the arch work in different running strike patterns; and 3) only male habitual runners were recruited in this study.

## 5 Conclusion

The 12-week gait retraining program combined with foot core exercise significantly increased the normalized navicular height during standing and the strength of arch muscles. During RFS running, such intervention decreased the maximum arch angle and increase arch height at touchdown, which indicated that the arch was improved in both static and dynamic positions. Furthermore, all of these significant changes had a moderate to large effect size, demonstrating the superiority of the combined intervention over the standalone interventions. Therefore, it is recommended that runners with weak arch muscles use this combined intervention as an approach to strengthen the arch muscles.

## Data Availability

The original contributions presented in the study are included in the article/supplementary material, further inquiries can be directed to the corresponding author.
